# Bioinformatics and In silico approaches to identify novel biomarkers and key pathways for cancers that are linked to the progression of female infertility: A comprehensive approach for drug discovery

**DOI:** 10.1371/journal.pone.0265746

**Published:** 2023-01-06

**Authors:** Md. Arju Hossain, Md Sohel, Md Habibur Rahman, Md Imran Hasan, Md. Sharif Khan, Md. Al Amin, Md. Zahidul Islam, Silong Peng

**Affiliations:** 1 Department of Biotechnology and Genetic Engineering, Mawlana Bhashani Science and Technology University, Tangail, Bangladesh; 2 Department of Biochemistry and Molecular Biology, Mawlana Bhashani Science and Technology University, Tangail, Bangladesh; 3 Department of Computer Science and Engineering, Islamic University, Kushtia, Bangladesh; 4 Department of Electronics, Graduate School of Engineering, Nagoya University, Nagoya, Japan; 5 Institute of Automation, Chinese Academy of Sciences, University of Chinese Academy of Sciences, Beijing, China; Universita degli Studi di Roma Tor Vergata, ITALY

## Abstract

Despite modern treatment, infertility remains one of the most common gynecologic diseases causing severe health effects worldwide. The clinical and epidemiological data have shown that several cancerous risk factors are strongly linked to Female Infertility (FI) development, but the exact causes remain unknown. Understanding how these risk factors affect FI-affected cell pathways might pave the door for the discovery of critical signaling pathways and hub proteins that may be targeted for therapeutic intervention. To deal with this, we have used a bioinformatics pipeline to build a transcriptome study of FI with four carcinogenic risk factors: Endometrial Cancer (EC), Ovarian Cancer (OC), Cervical Cancer (CC), and Thyroid Cancer (TC). We identified FI sharing 97, 211, 87 and 33 differentially expressed genes (DEGs) with EC, OC, CC, and TC, respectively. We have built gene-disease association networks from the identified genes based on the multilayer network and neighbour-based benchmarking. Identified TNF signalling pathways, ovarian infertility genes, cholesterol metabolic process, and cellular response to cytokine stimulus were significant molecular and GO pathways, both of which improved our understanding the fundamental molecular mechanisms of cancers associated with FI progression. For therapeutic intervention, we have targeted the two most significant hub proteins VEGFA and PIK3R1, out of ten proteins based on Maximal Clique Centrality (MCC) value of cytoscape and literature analysis for molecular docking with 27 phytoestrogenic compounds. Among them, sesamin, galangin and coumestrol showed the highest binding affinity for VEGFA and PIK3R1 proteins together with favourable ADMET properties. We recommended that our identified pathway, hub proteins and phytocompounds may be served as new targets and therapeutic interventions for accurate diagnosis and treatment of multiple diseases.

## Introduction

In modern civilization, infertility is a significant threat globally, including in developing and developed countries. Infertility, according to Zegers-Hochschild et al. is the result of a malfunction in the reproductive system. There are two types of infertility in women: primary and secondary. Women with primary infertility have never been clinically diagnosed with pregnancy, but women with secondary infertility could not develop a pregnancy clinically even before being diagnosed [1]. Secondary infertility is more frequent than primary infertility for women [1–3] Inhorn (2014) reported that the prevalence of infertility up to 186 million people around the world with more incidence rate was confined to developing countries [4], including South Asia and Central Asia, Central and Eastern Europe, North Africa and the Middle East, and some regions of sub-Saharan Africa [1]. The women’s infertility rate was calculated at around 8 percent, 13-14 percent, and 18 percent at the age of 19-26, 27-34, and 35-39 years, respectively [5]. Although the exact etiology of infertility in women was unknown, generic and cancerous risk factors might be associated with infertility. Ovarian cancer is one of the fatal of all gynecological diseases worldwide. Most commonly, two mutated genes, including breast cancer 1 (BRCA1) and breast cancer 2 (BRCA2) who were inherited, causing ovarian cancer and breast cancer in women [6]. Cirillo et al. found a linkage between ovarian cancer and irregular menstruation in their study [7]. Cervical cancer is the world’s fourth most prevalent disease among females [8]. According to global report in 2018, approximately 570,000 patients were diagnosed with cervical cancer and 31,000 were died [9]. The study by J. Dor et al. mentions that cervical cancer has been linked to infertility-causing pelvic infections or adhesions in the past [10].

Endometrial cancer (EC) is the fifth most common cancer in women from developed countries, accounting for 4.8 percent of new cases and 2.1 percent of deaths [11]. Autosomal dominant mutations cause this disease in DNA mismatch repair (MMR) genes [12]. Patients who have a germline mutation in the MMR gene have a 20–70 percent lifetime risk of developing EC, depending on their individual circumstances [13]. Women who have endometriosis are more likely to have infertility, according to a study conducted by Bulun et al. [14]. The most frequent endocrine malignancy is thyroid cancer (TC) accounts for 3.4 percent of all malignancies diagnosed each year [15]. Thyroid cancer is caused by genetic and epigenetic changes, including mutations in genes of BRAF, RAS, PIK3CA, PTEN and so on [16]. Menstrual disorder and an increased risk of miscarriage in infertile women due to thyroid abnormalities are more frequent [17]. These mentioned risk factors can influence female infertility when they commonly share dysregulated gene expression (DEG) [18], and the molecular pathways that may trigger the influential factors to promote infertility and cancer can be exposed by analyzing protein-protein interaction (PPI), metabolic pathway and gene ontologies [19].

Additionally, several pathways may correlate important genes involved with the progression of infertility to various types of cancer. PIK3R1 is a p85 regulatory protein encoded by the Phosphatidylinositol-kinase regulatory subunit alpha gene that regulates the p110 catalytic subunit [20], where most frequent mutation occurs in ovarian cancer [21] and endometrial carcinomas [22], within the iSH2 domain. So, PIK3R1 maybe act as a therapeutic target for infertility and infertility-related cancer. On the other hand, VEGFA can also be a therapeutic target in various infertility mediated gynecological cancer like ovarian [23] and cervical cancer [24].

Network pharmacology is a new approach that combines computer science and medicine by building and visualizing a “multi-gene, multi-target, multi-pathway interaction network” to assess the drug’s molecular mechanism [25]. The molecular docking method means that a small molecule is spatially attached to a macromolecular system and can detect the additional value at bindings used in structural drug design [26]. Researchers have recently made many integrative network analysis approaches to classify biomolecules’ potential functions in other diseases [27, 28].

However, genetic experiments were carried out to study the effect of risk factors on female infertility, but network-based approaches were not implicated for such type of study [29]. Integrative research is crucial for understanding and identifying the disease-causing molecular pathways. Therefore we aimed to use a network-based bioinformatics pipeline to elucidate the cancerous risk factors and genes of female infertility; those mediate disease progression through gene expression profiling, metabolic pathway analysis, gene ontologies, and PPI sub-network interaction analysis. Moreover, we targeted hub proteins from PPI analysis for molecular modelling and ADMET analyses with 27 phytoestrogenic compounds for therapeutic intervention. Finally, we have used gold benchmark databases OMIM and DisGeNET, as well as literature to validate the known FI associated genes and molecular pathways.

## Materials and methods

### Details information of GEO datasets

In our study, five different microarray datasets were analyzed, including Female Infertility (FI), Endometrial Cancer (EC), Ovarian Cancer (OC), Cervical Cancer (CC), and Thyroid Cancer (TC) with the accession numbers GSE92324, GSE63678, GSE124766, GSE29570, and GSE6004, respectively from the National Center for Biotechnology Information (NCBI) Gene Expression Omnibus (GEO) database. Table 1 provides a summary of the information contained in the datasets.

**Table 1:** An overview of datasets with their geo-features and quantitative measurements is provided.

GSE number	Disease name	Source name	Control sample	Case sample	DEGs	Up regulated genes	Down regulated genes	References
GSE92324	Female Infertility	endometrial tissue	8	10	1201	651	550	[46]
GSE63678	Endometrial Cancer	endometrial tissue	5	7	723	456	267	[47]
GSE124766	Ovarian Cancer	ovarian tissue	8	20	2101	1045	1057	[48]
GSE29570	Cervical Cancer	biopsy tissue	17	45	880	535	345	[49]
GSE6004	Thyroid Cancer	frozen surgical tissue	3	12	541	338	203	[50]

### Data preprocessing and identification of DEGs

Based on microarray data, global transcriptome analysis was implicated in investigating the FI’s gene expression profiles with four cancerous risk factors to determine the molecular characteristics of human disorders. As different errors are typically accounted for in the preparation and analysis of microarray data in our study. In each sample, the disease group or control group must be standardized. One of the most common methods for standardizing gene expression matrices is the Z-score transform [30]. If *X*_*ij*_ is the expression value of the i-th gene in sample j, then Z-score transform standardization is obtained as follows:

$$\begin{array}{c} Z_{ij}=\frac{X_{ij}-X_{i}}{\sigma_{i}} \end{array}$$ (1)

Where *σ*_*i*_ and *X*_*i*_ are considered the standard deviation and mean of the expression value of the i-th gene expected inclusive samples, respectively. This transformation enables a clear comparison of the values of gene expression in various models and diseases. We have conducted a linear regression technique on data from a time series and this Z-score transformation for achieving a joint t-testing statistic between two groups. Data were converted into log2, and the linear regression model was employed to compute expression levels of each gene through the following formula:

$$\begin{array}{c} Y_{i}=\beta_{0}+\beta_{1}X_{i} \end{array}$$ (2)

Here, *Y*_*i*_ is the gene expression value, and *X*_*i*_ is the disease state in this case (disease or control). The model parameters *β*_0_ and *β*_1_ were calculated applying least squares.

In this study, we first compared diseased tissue against controls to identify differentially expressed genes (DEGs) associated with their respective pathology. For clarification, the inclusion criteria of a study subject might be female groups between the ages of 21 to 45 who have been diagnosed with different stages of diseases. Exclusion criteria for this study might be cell line data and male groups for thyroid cancers. To make consistent expression data from different platforms and avoid the problems of experimental systems, we normalized the gene expression data comprising disease state and control data by using the quantile normalization and Z-score transformation techniques through the NCBIs GEO2R online tool. We performed the analysis of the microarray data using well established Linear Models for Microarray Data (LIMMA). Then we used an unpaired t-test in which essential genes were selected to see if any genes are differentially expressed in disease and control by setting P-value < 0.05 and an absolute value of |*logfold* − *change*| > 1. Moreover, a two-way analysis of variance (ANOVA) test was performed to determine the statistical significance between groups. P-values were adjusted by the well-established Benjamini-Hochberg method and Bonferroni correction method as indicated. Based on varying the False discovery rate (FDR) threshold and standard statistical criteria; we considered P-value< 0.05 and |*logfold* − *change*| ≥ 1 for up-regulated genes, while P-value<0.05 and |*logfold* − *change*| ≤−1 for finding downregulated genes. Then we have provided the rationale and justification for the selection of common DEGs by hypergeometric tests. We performed hypergeometric tests for the DEGs to establish their role as predictive diagnostic biomarkers for female infertility and four selected cancers represented in Table 2. Further, to determine the shared DEGs, we compared the FI dataset with four other selected diseases using Venny v2.1 web tool [31]. Then the gene-disease network (GDN) was created and visualized with Cytoscape v3.8.2 [32].

**Table 2:** Validation of DEGs by hypergeometric test.

Disease Pair	Female infertility DEGs	Cancer DEGs	Common DEGs	Hypergeometric p-value	Representation factor
FI+OC	1201	2101	211	1.531e-53	3.3
FI+EC	1201	723	97	6.089e-36	4.5
FI+CC	1201	880	87	2.316e-22	3.3
FI+TC	1201	541	33	5.293e-05	2.1

### Pathway and functional enrichment analysis

The signaling pathways and gene ontology of FI were assessed using the web-based gene set enrichment analysis tool EnrichR for all the genes that were differentially expressed in FI and cancerous risk factors [33]. The research included selecting gene ontology (GO) (biological processes, molecular function and cellular component) and signalling pathways (KEGG, WiKi, and Reactome) as an annotation source [34]. For statistical significance, the adjusted p-value was considered to achieve enhancement results.

### Protein interactome analysis

For the assembly and study of the network protein-protein interaction, the web-based visualization software STRING has been employed, and Cytoscape (v3.8.2) was used for further evaluation [32, 35, 36]. The PPI network used a graph with no direction for representation, where the nodes denoted the proteins, and the edges indicated the proteins’ interactions. To classify strongly interconnected proteins (i.e., hub proteins), we conducted a topology study applying the Cyto-Hubba plugin, and the Maximal Clique Centrality (MCC) algorithm was implicated.

### Protein and ligand preparation

The two proteins, VEGFA (PDB ID: 1FLT) and PIK3R1 (PDB ID: 5M6U) were prepared by retrieving the three-dimension crystal structure from RCSB PDB [36–38]. The 3D structure of target proteins was prepared by removing water through Discovery Studio (Studio 2015), and Pymol [39] software package and minimized energy with GROMOS96 43B1 force field through SWISS PDB Viewer [40]. We prepared a phytoestrogen dataset from previous experimental studies searching related literature in PubMed, Scopus databases, the web of science, Google scholar; those were used as significant compounds in several cancer treatments. Then SDF format of all ligand molecules was retrieved from the PubChem database [41]. Pymol Software was used to convert SDF format compounds to PDB format. For optimization and preparation of ligands, we used PyRx integrated mmff94 (Merck molecular force field) [42, 43].

### Virtual screening

Molecular docking by AutoDock wizard has been done to understand the link between the ligand and the drug compounds with PyRx. Ligand and target protein was considered as flexible and rigid, respectively, during ducking. Ligands with the lowest RMSD values and the highest negative docking scores were considered for ADMET evaluation. Finally, Discovery studio and Pymol tools were used to examine the docked pose for molecular interactions between ligands and receptors.

### ADMET analysis

Compounds with higher binding affinity were selected for the ADMET study. SwissADME was used to measure the Absorption, Distribution, Metabolism, and Elimination (ADME) properties of potent drug candidates by PubChem provided canonical SMILES of selected ligands [44], while pkCSM toxicity prediction tools were used to investigate toxicity [45].

### Overview of a proposed integrated pipeline

**Fig 1:**
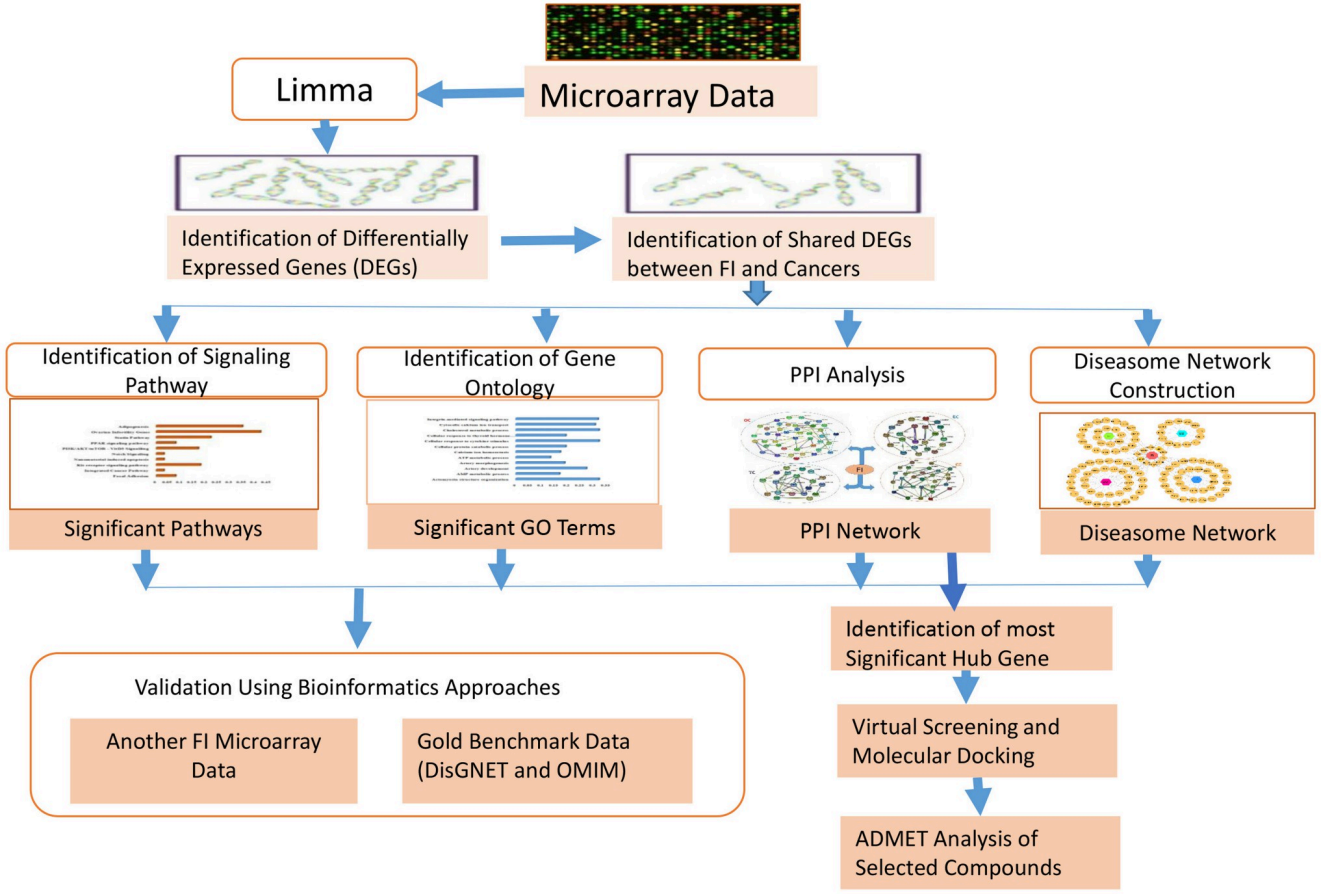
Block diagram of the proposed bioinformatics and system biology pipeline.

This research work developed and applied a multi-step quantitative approach as an integrated pipeline of bioinformatics and molecular docking approaches methodologies, as shown in Fig 1.

## Results

### Gene expression analysis

**Fig 2:**
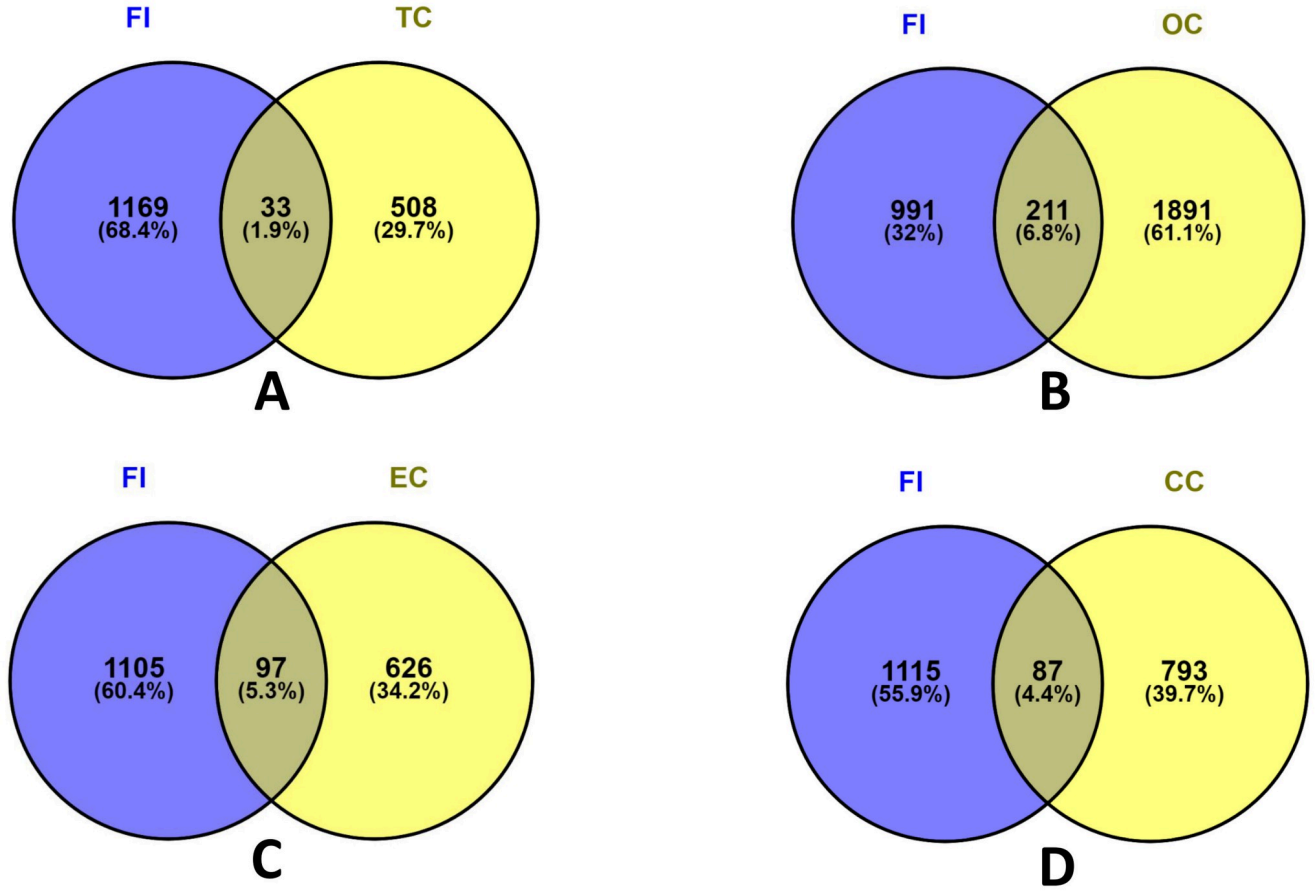
Venn diagram was applied to present all candidate targets of Female Infertility (FI) and four cancerous risk factors, where FI shared a common dysregulated gene between A. Thyroid cancer (TC), B. Ovarian cancer (OC), C. Endometrial cancer (EC), and D. Cervical cancer (CC).

**Fig 3:**
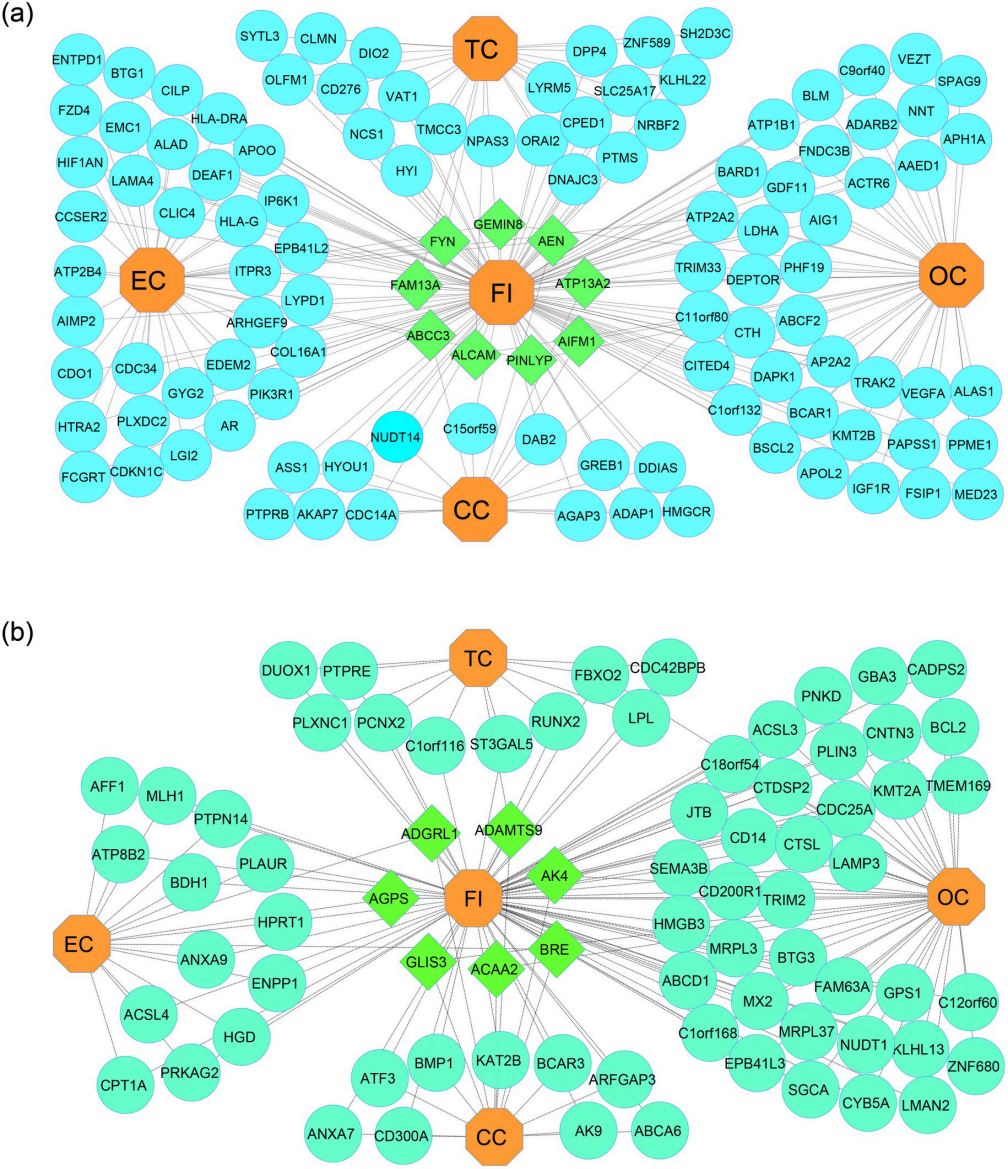
**a**: Gene-Disease network of common DEGs having upregulated genes between Female Infertility (FI) with Endometrial Cancer (EC), Ovarian Cancer (OC), Cervical Cancer (EC), and Thyroid Cancer (TC). Octagon-shaped and light red color nodes represent four risk factors, while round-shaped and sky color nodes represent DEGs. Square-shaped and green color nodes indicate DEGs are common among FI and four risk factors. **b**: Gene-Disease network of common DEGs having down-regulated genes between Female Infertility (FI) with Endometrial Cancer (EC), Ovarian Cancer (OC), Cervical Cancer (EC) and Thyroid Cancer (TC). Octagon-shaped and light red color nodes represent four risk factors, while round-shaped and sky color nodes represent DEGs. Square-shaped and green color nodes indicate DEGs are common among FI and four risk factors.

The genetic variation patterns from endometrial tissues were used to identify dysregulated genes linked to FI during implantation failure cases of infertile patients were investigated and differentiated with the standard subject [46] by using R Bioconductor package Limma through NCBI GEO2R online tool. Compared to normal subjects, 1201 genes were differentially expressed (651 genes upregulated and 550 genes down-regulated). To investigate the relationship between the FI transcriptome and each risk factor, we implemented mRNA microarray data through a series of cross comparative analyses. The Venn diagram of Fig 2 shows that FI shared 97, 211, 87 and 33 genes with EC, OC, CC, and TC, respectively. Using Cytoscape, a gene-disease relationship network (GDN) based on FI data were created to find statistically meaningful associations among these risk factors. The relation between over and under-expressed genes is represented through the networks shown in Fig 3a and 3b. The most critical DEGs are defined using our proposed method, summarized in Table 1.

Three fundamental genes were discovered in our research, including ABCC3, AEN, and ADAMTS1 are generally over-expressed among the FI, CC, and EC; ATP13A2 and AIFM1 are two upregulated genes found in the FI, CC, and OC; two crucial genes ALCAM and FAM13A are frequently upregulated in FI, TC, and OC. Three important genes PINLYP, GEMIN8, and ATP2A2 are associated with FI, EC, and OC.

On the other hand, the three down-regulated AK4, ACAA2, and GLIS3 genes are prevalent in the FI, CC, and OC; Two under-expressed genes BRE and ADGRL1 are shared in the FI, CC, and EC. The FI, TC, and CC are typical for one down-regulated gene ADAMTS9; one down-regulated FBXO2 gene is expected in the FI, OC, and TC. Besides, FI, OC, and EC are frequently shared one down-regulated gene AGPS.

### Pathway and functional association analysis

**Fig 4:**
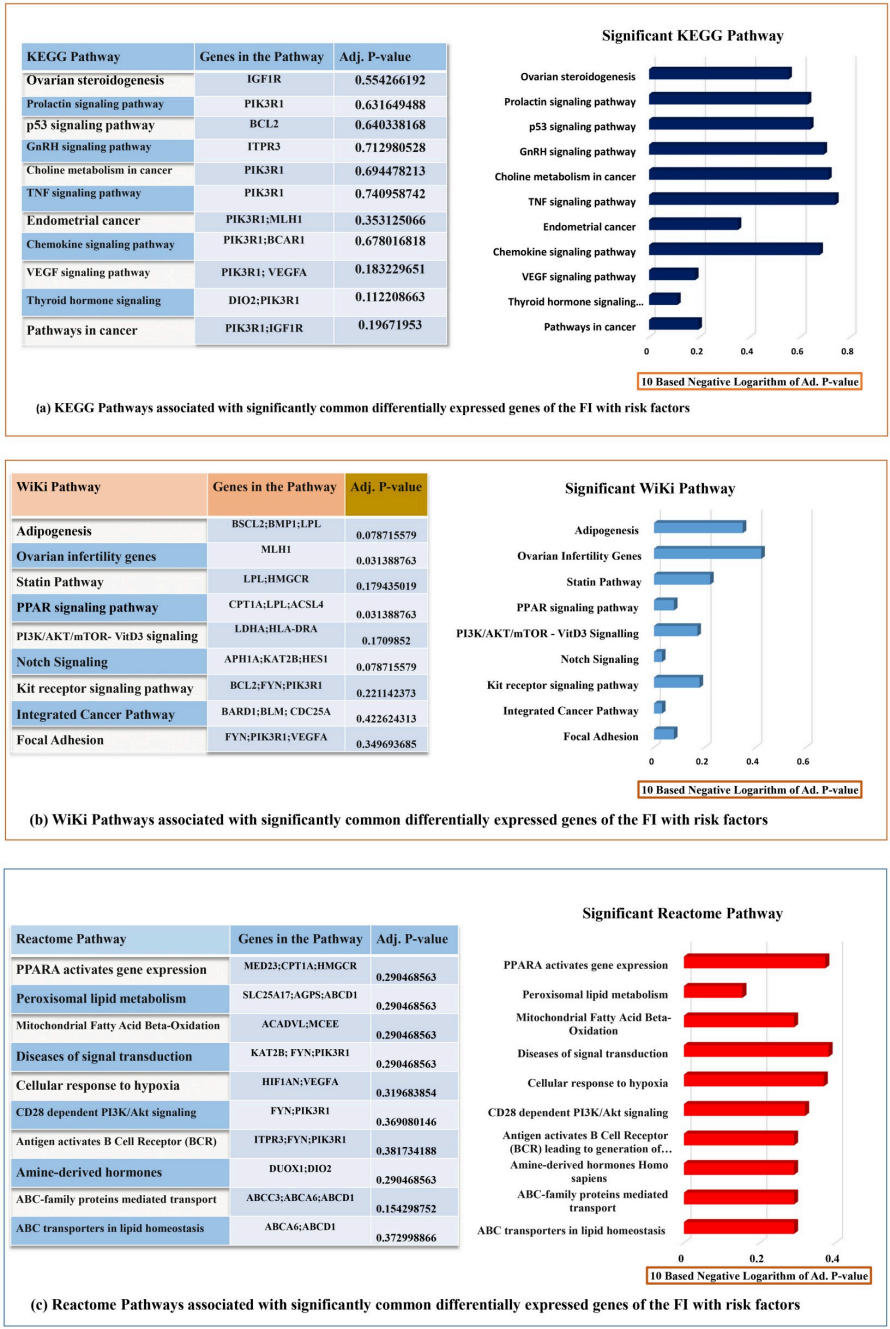
**a**: The top 20 signalling pathways from KEGG enrichment analysis were showed by the bar diagram with p-values. **b**: The top 20 signalling pathways from WiKi enrichment analysis were showed by the bar diagram with p-values. **c**: The top 20 signalling pathways from Reactome enrichment analysis were showed by the bar diagram with p-values.

EnrichR online platform uses all differentially expressed common genes to find significant molecular pathways linked to FI and four risk factors through KEGG, WiKi, and the Reactome pathway database. The enrichment study identified 234, 253, and 102 pathways among KEGG, WiKi, and Reactome databases, respectively. Particularly, we considered only ten significant pathways of each pathway database after p-value adjustments associated with the cancer progression. The most considerable pathways have been found which are Proteoglycans in cancer (hsa05205), Notch Signaling (hsa04330), PPAR signalling pathway (hsa03320), Pathways in cancer (hsa05200), Diseases of signal transduction (R-HSA-5663202), CD28 dependent PI3K/Akt signalling (R-HSA-389357), ABC transporters in lipid homeostasis (R-HSA-1369062), VEGFA-VEGFR2 Signaling Pathway (hsa04370), and Thyroid hormone signalling pathway (hsa04919). These mentioned pathways and other significant mutual pathways are given in Fig 4a–4c.

**Fig 5:**
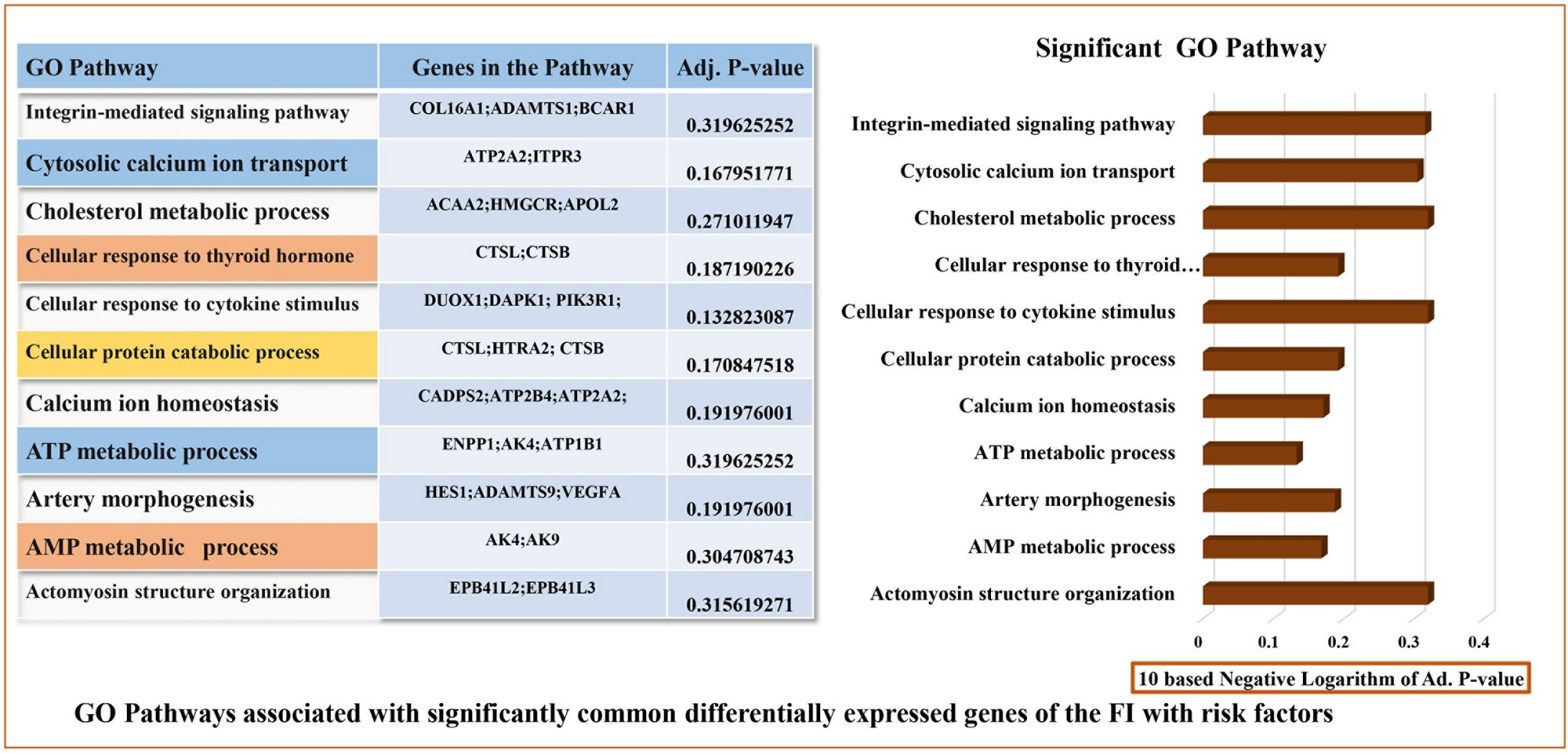
The top 20 signalling pathways from GO biological process enrichment analysis were showed by the bar diagram with p-values.

We used an ontology enrichment study to find 1586 GO terms (biological processes) for the commonly dysregulated genes among FI and cancerous risk factors. The primary significant GO classes are actomyosin structure organization, integrin-mediated signalling pathway, cholesterol metabolic process, ATP metabolic process, and cellular response to cytokine stimulus depicted in Fig 5.

### Establishment protein-protein interaction network

**Fig 6:**
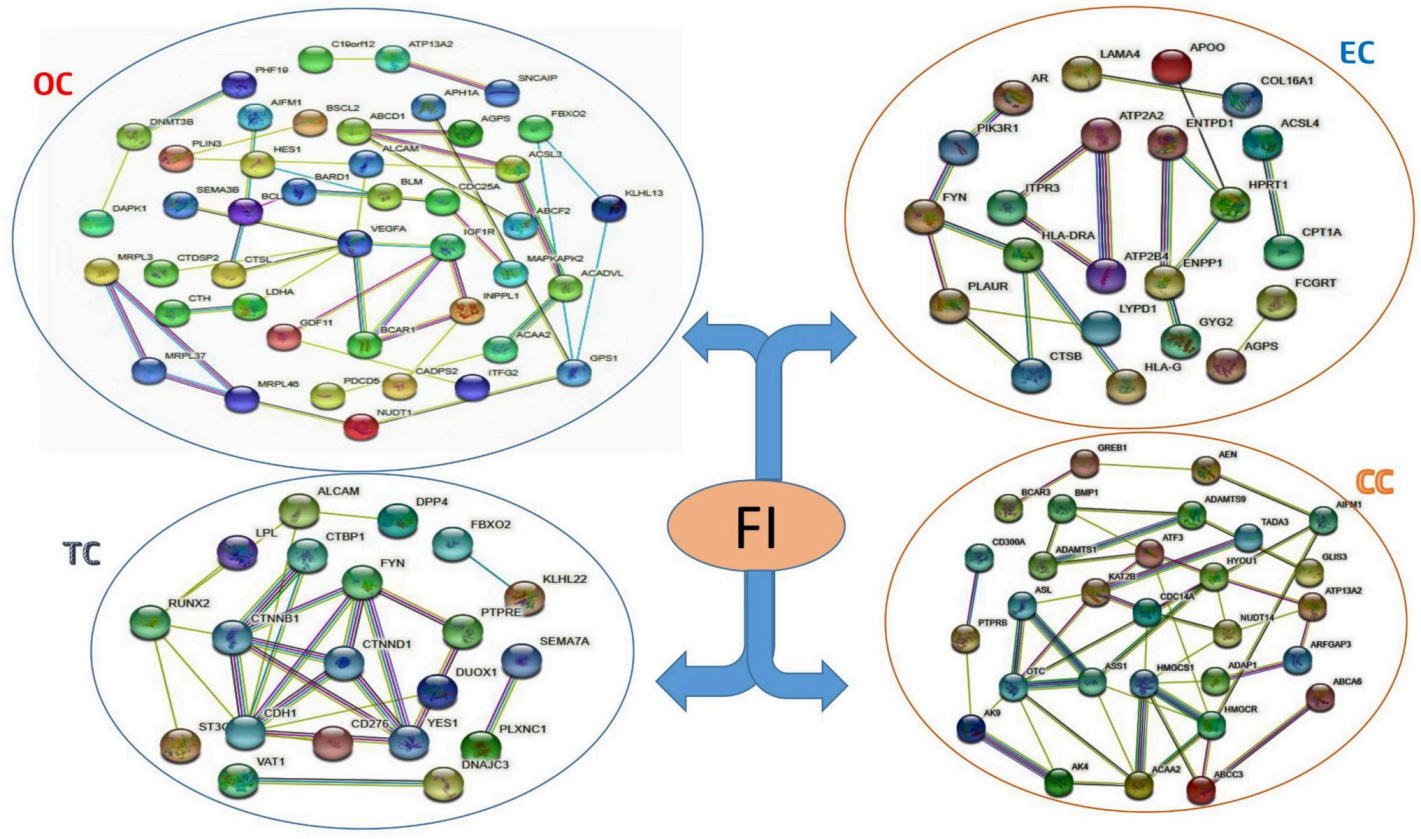
Protein-Protein interaction network for the common paired DEGs between Female Infertility (FI) and Endometrial Cancer (EC), Ovarian Cancer (OC), Cervical Cancer (EC), and Thyroid Cancer (TC). The network nodes depict target proteins, and the edges represent protein-protein relationships.

Both differentially expressed genes found in the FI and other risk factors to build the PPI network are shown in Fig 6. Each node in the network represents a protein and an edge represents the connection between two proteins. In addition, the network is split into four clusters, each of which represents a risk factor.

**Fig 7:**
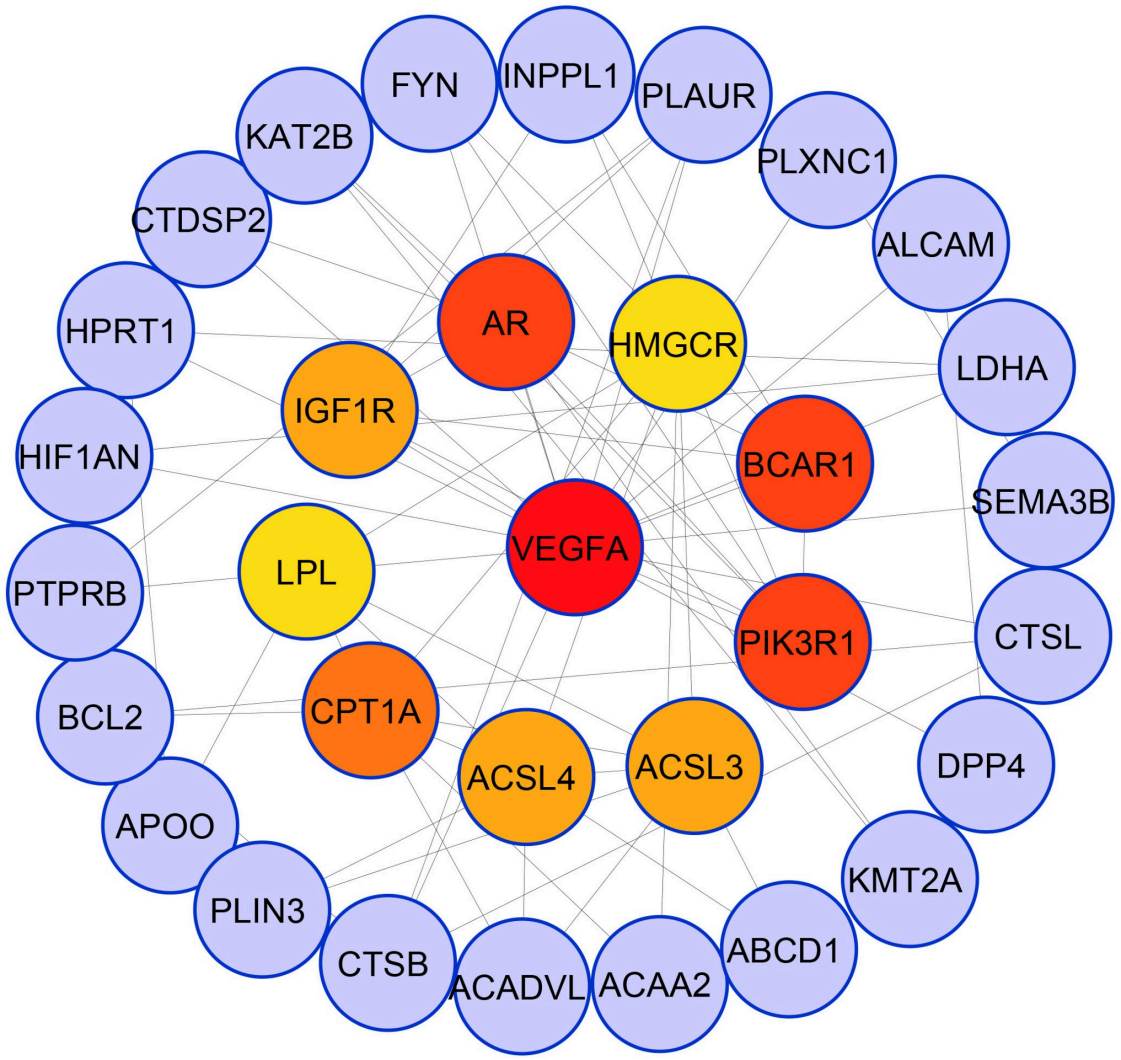
The simplified PPI network depicting the hub proteins. The ten most significant hub proteins are ranked with red to the yellow-colored gradient.

Using the Cyto-Hubba plugin, a generalized PPI network was created for topological analysis [61], displaying the ten most essential hub proteins in Fig 7: VEGFA, PIK3R1, BCAR1, AR, CPT1A, ACSL3, ACSL4, IGF1R, LPL, and HMGCR. Interestingly, each of the RUNX2, BCAR1, VEGFA, and PIK3R1 proteins belongs to one of the four clusters, suggesting that the FI shares them and the other three risk factors have interacted with in various clusters by other protein. On the other hand, BCL2 and ITPR3 belong to three groups and interrelate with other proteins in the network.

Four out of the ten hub proteins are dysregulated due to OC; EC dysregulates four, and two proteins are dysregulated by CC and TC. For docking purposes, these hub proteins may be the target proteins. The functionally significant ten hub proteins with molecular function were tabulated in Table 3.

**Table 3:** Molecular functions of the ten hub proteins associated with the four risk factors.

Protein name	Description	MCC value	Molecular function	Ref.
**VEGFA**	Vascular endothelial growth factor A	58	Responsible for tumor growth and angiogenesis	[51]
**PIK3R1**	Phosphoinositide-3-Kinase Regulatory Subunit 1	57	Underexpression may cause PI3K pathway activation and contribute to growth and progression	[52]
**BCAR1**	Breast cancer anti-estrogen resistance protein 1	38	Overexpression performs a key role in carcinogenesis, most likely via activating the p38 MAPK pathway.	[53]
**AR**	Androgen receptor	38	Stimulates cell proliferation prostate cancer via regulating the G1/S transition in the cell cycle	[54]
**CPT1A**	Carnitine Palmitoyltransferase 1A	35	CPT1A expression is required for adipocytes to promote tumor growth and initiation	[55]
**ACSL3**	Acyl-CoA Synthetase Long-Chain Family Member 3	34	Overexpression of ACSL3 enhanced tumor cell proliferation, migration, and invasion, all of which favoured malignancy.	[56]
**ACSL4**	Acyl-CoA Synthetase Long Chain Family Member 4	34	Increase uncontrolled cell proliferation, facilitate tumor invasion and evade programmed cell death	[57]
**IGF1R**	Insulin Like Growth Factor 1 Receptor	34	Regulates cell proliferation and survival	[58]
**LPL**	Lipoprotein Lipase	28	Plays important roles in inflammation, obesity and cancer development	[59]
**HMGCR**	3-Hydroxy-3-Methylglutaryl-CoA Reductase	28	Positively regulates the growth, migration, and tumorigenesis of several cancer cells	[60]

### Virtual screening of retrieved compounds against VEGFA and PIK3R1 proteins

**Fig 8:**
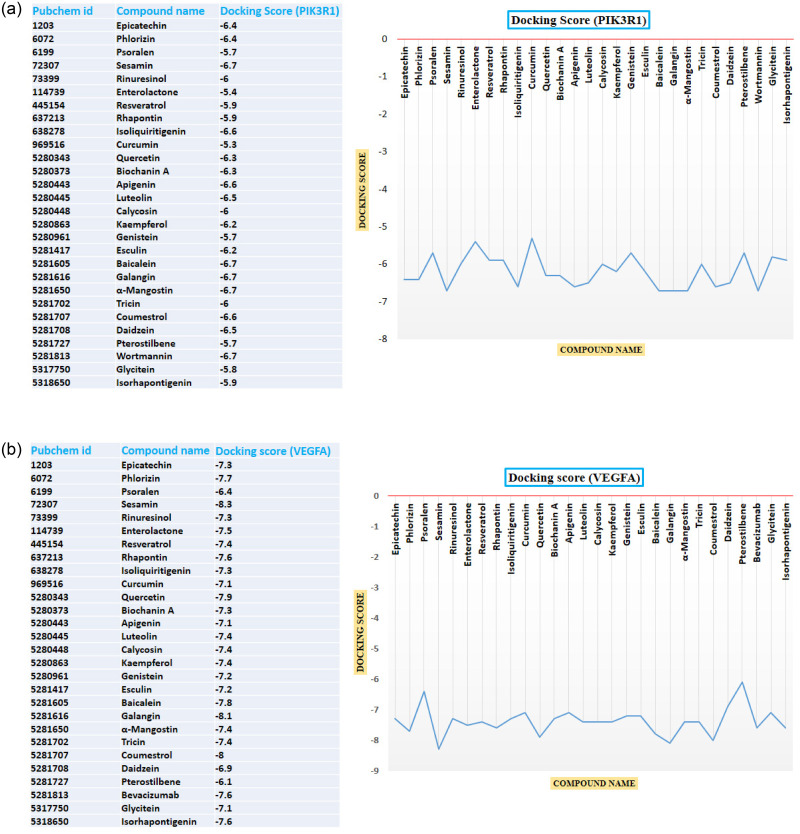
**a**: Docking scores of all compounds. Most of the compounds have a range between -5 to -7 kcal/mol binding affinities. **b**: Docking scores of all compounds. Most of the compounds have a range between -5 to -8.3 kcal/mol binding affinities.

Virtual skimming with molecular docking is another technique to identify a lead compound in the drug discovery process. In our study, we took ten proteins with their rank of significance by cyto-Hubba plugin analysis. The significant protein, VEGFA and PIK3R1, were selected to conduct molecular docking purposes based on two criteria. First, maximal clique centrality (MCC) algorithm of Cytohubba plugin, VEGFA and PIK3R1 protein in a ranking of first and second position respectively which indicate most two significant proteins. Second, by searching the literature, we found VEGFA and PIK3R1 protein are the most interconnected among our selected cancer type risk factors, including Endometrial cancer [62, 63], Ovarian cancer [23, 64], Cervical cancer [24, 65] and Thyroid cancer [66, 67]. Then, a total of 27 phytoestrogenic compounds with control were chosen for molecular docking, showing an anti-cancer activity through literature analysis. Based on binding affinity, four compounds, including sesamin, alpha-mangostin, galangin, coumestrol, and quercetin, are considered for further analysis. This research used bevacizumab and wortmannin as a positive control ligand for VEGFA and PIK3R1 proteins, respectively. Wortmannin was a potent inhibitor (binding affinity -6.8 kcal/mol) against PIK3R1 protein that encodes P85 regulatory subunit, which regulates the P110 catalytic subunit in inter-Src homology-2 (iSH2) domain to the plasma membrane. Besides, bevacizumab (binding affinity -5.5 kcal/mol) can be used as a first approved angiogenesis inhibitor via VEGFA targeting protein [68]. The selected compounds with compound names and binding affinity are given in Fig 8a and 8b.

### Molecular interaction analysis of selected compounds

The active site of VEGFA (PDB ID: 1FLT) and PIK3R1 (PDB ID: 5M6U) proteins were predicted using CASTp server [69]. The domain part of PIK3R1 (chain B) protein is inter-Src homology-2 (iSH2) provided 400 to 600 amino acid sequence identified through Interpro server [70, 71]. Several frequent mutations in the inter-Src homology-2 (iSH2) domain, including Y504D, Q552K, I559N, D560Y, N564D, D569Y, R574T, T576del, W583del, N595K, and N600H mutants may disrupt the inhibitory interaction of the C2 domain with iSH2 and this residue act as a hotspot to occur mutation for oncogenesis like breast, endometrium and ovarian cancer particularly at amino acids 456-469 and 564-575 [70, 72–74]. The role of some functionally important mutated amino acids in the iSH2 domain were tabulated in Table 4 which are responsible for several tumor progression. In our investigation, we did not include the mutated form of PIK3R1 protein. In addition, all the selected phytochemicals bind to the only active site of this protein, and no one ligand can be bound to mutate amino acids. Our result demonstrated that the amino acid position 436-599 is expected to be conserved in the PIK3R1 protein active site. In a VEGFA protein, the domain site has resided in 39 to 135; a conserved site is found in 75 to 87 amino acid positions. Therefore, in our study, the docked compounds interaction showed that all compounds interact same binding pocket with the identical catalytic residues, including Gln 497, Ser 505, Tyr 508, Ile 524, Asn 527, and Tyr 528 for PIK3R1 protein (Table 5), while Glu 64, Ser 50, Cys 68, Ile 46 for VEGFA protein (Table 6). The ligand forms interaction with substrate-binding pocket residues were visualized using the BIOVIA discovery studio visualizer.

**Table 4:** Role of some functionally essential mutated amino acids in the iSH2 domain.

Protein name	Mutant form	Function
PIK3R1	N564D	to increase PI3K activity in endometrial tumors as a positive control
	R574T and T576del	to enhance roughly cell growth for oncogenic transformation
	G560Y	to disrupt the helix or change the length of the iSH2 domain as a single mutant
	E542K and E545K	contribute to various aspects of tumor development via regulating cellular pathways

**Table 5:** Docking simulation results of 4 compounds with docking score energy and interaction with amino acids against PIK3R1 protein.

Compound Name	Docking energy (kcal.mol-1)	Hydrogen Bond Ligand atom-Amino Acid Distance (Å)	Hydrophobic Bond Interaction-Amino Acid Distance(Å)
Sesamin	-6.7	SER505:HG(2.99288)	Pi-Pi T- shaped-TYR528 (4.79069)
		GLU502:HA(2.58332)	Pi-Alkyl—LYS506 (3.77723)
		GLU502: O (2.34831)	
		GLU502:OE2 (3.06726)	
*α*-mangostin	-6.7	SER505:HG(2.51696)	Pi-Pi T-shaped -TYR528(4.7623)
		SER505:HB2(2.66301)	Pi-Pi T-shaped -TYR528(5.12308)
			Alkyl—ILE509(5.44374)
			Alkyl—MET525(4.67183)
			Alkyl—LYS506(4.17836)
			Alkyl—ILE509(5.41536)
			Alkyl—LYS532 (4.48014)
Galanzin	-6.7	GLN497:HE21(2.26174)	Pi-Pi T-shaped—TYR508(4.75895)
			Pi-Alkyl—ILE524(4.34365)
Coumestrol	-6.6	GLN501:HA (2.89717)	Pi-Pi T-shaped—TYR508(4.75166)
		SER505:HB1(2.96075)	Pi-Alkyl—ILE524(4.51341)
			Pi-Alkyl—ILE524(5.34046)
			Pi-Alkyl—ILE524 (4.21717)
Wortmannin	-6.8	GLN497:HE21(2.82878)	Pi-Pi Stacked—TYR504(4.64417)
		ASN527:OD1(3.04664)	Pi-Alkyl—TYR504(5.17453)
		GLN497:HE21(2.82878)	
		ASN527:OD1(3.04664)	

**Table 6:** Docking simulation results of 4 compounds with docking score energy and interaction with amino acids against VEGFA protein.

Compound Name	Docking energy (kcal.mol-1)	Hydrogen Bond Ligand atom-Amino Acid Distance (A)	Hydrophobic Bond Interaction-Amino Acid Distance(A)
Bevacizumab (Control)	-5.5	Y:ARG133:HH11 (2.31056)	Y:TYR139 (3.80127)
		Y: LYS217:HN (2.29829)	V: LYS16 (4.51013)
		Y:TYR139:OH (2.3838)	V:MET18 (5.29144)
		Y:LEU215:O (2.6249)	V:MET18 (4.84131)
			Y: TYR139 (4.87068)
			Y: LYS217 (4.78722)
Coumestrol	-8	W: GLU64:HN (2.10073)	W: GLU64:OE2 (4.70255)
		W: ASP63:HA (2.67885)	W: GLU64:OE2 (4.81957)
		X:THR226:HA (2.96673)	V:ILE46 (3.85383)
		V:PHE47:HN (2.86934)	V:ILE46 (3.62054)
			V: ILE46 (5.07809)
			V: ILE46 (4.35264)
Galangin	-8.1	V: CYS61:HN (1.98732)	V: GLU64:OE1 (4.51529)
		V: CYS68:HN (2.11427)	
		V: CYS60:HA (2.50449)	
		V: GLU67:HA (2.53521)	
		W: SER50:HG (2.86959)	
Quercitin	-7.9	W:ASN62:O (2.9349)	W:ASP63:OD1 (3.75656)
		V:ASP34:OD2 (1.99649)	V:ILE46 (1.99649)
		V:TYR45:O (4.98128)	V:ILE46 (4.98128)
		V:PHE36:HN (3.19653)	
		V: SER50:HG (2.56624)	
		V: SER50:HB1 (4.77306)	
		V: PHE47:HN (2.9349)	
		W: GLU64:HN (2.73851)	
Sesamin	-8.3	V: CYS61:HN (2.47451)	V: GLU64:OE1 (4.20541)
		V:GLY59:O (2.54908)	C- W:ILE46 (4.22532)
		V:CYS57:O (2.9038)	C- V:CYS68 (4.53856)
		V:CYS68:O (2.9161)	V:CYS68 (4.92696)
		V: CYS57:O (3.08045)	
		V: CYS68:HN (2.9202)	
		W: SER50:HG (2.63393)	

**Fig 9:**
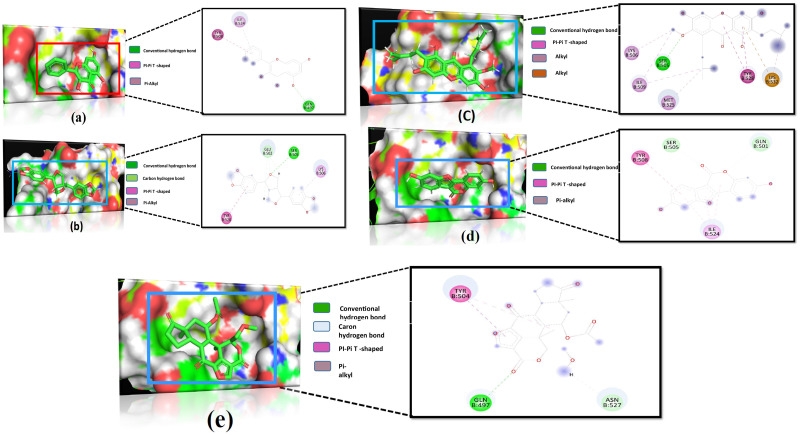
Molecular interactions analysis of selected compounds against PIK3R1 (PDB ID: 5M6U) of (a) galangin, (b) sesamin, (c) alpha-mangostin, (d) coumestrol and (e) control.

**Fig 10:**
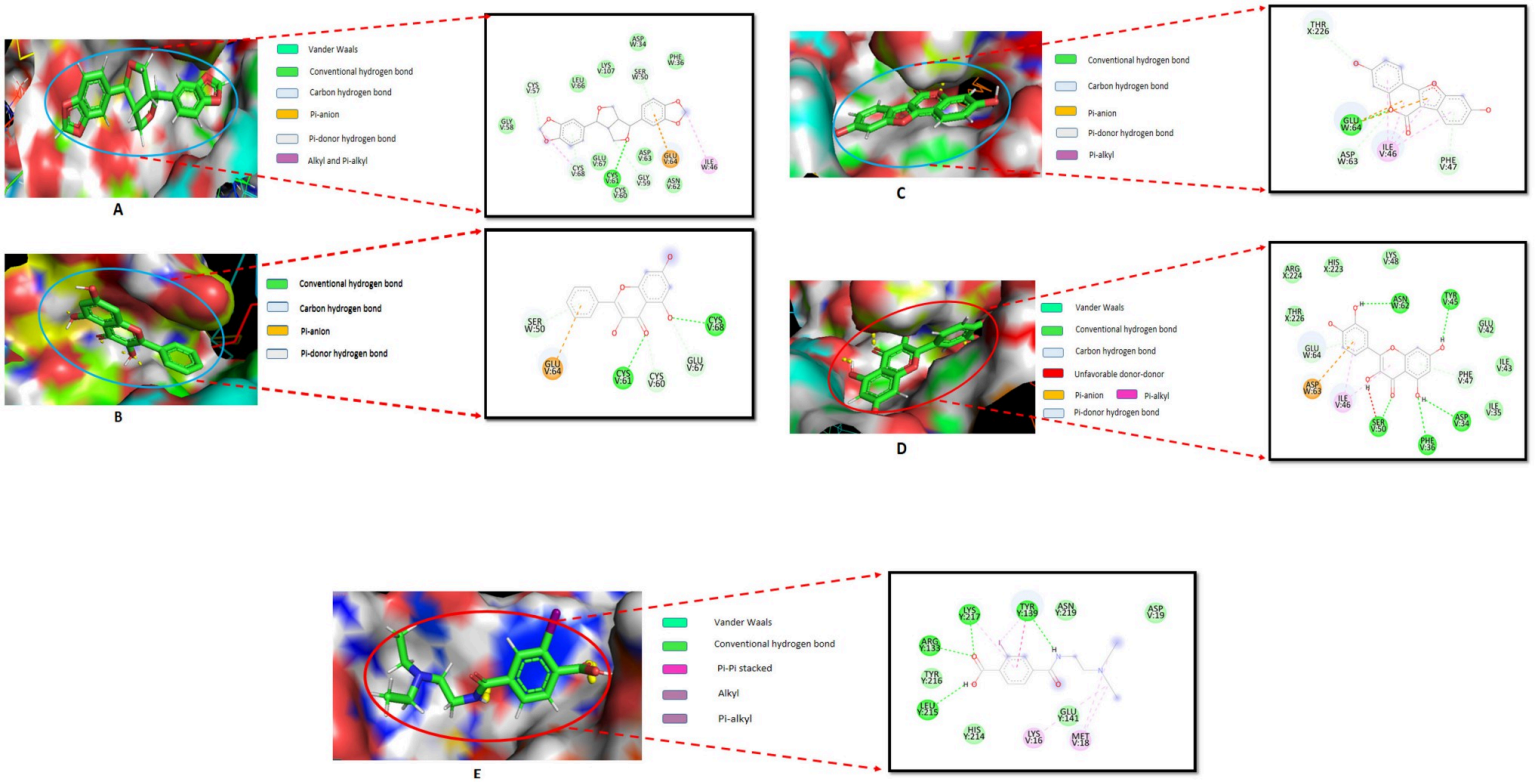
Molecular interactions of selected compounds against VEGFA (PDB ID: 1FLT) of (A) sesamin, (B) galangin, (C) coumestrol, (D) quercetin and (E) control. All selected compounds interact with the vital substrate management catalytic site.

Sesamin formed two hydrogen bonds at Glu 502, Ser 505, and two hydrophobic bonds at Lys 506 and Tyr 528. On the contrary, alpha -mangostin showed one hydrogen bond at Ser 505 and one pi-pi-T shaped at Tyr 528, four alkyl bonds at Lys 506, Ile 509, Met525 and Lys 532. Besides, PIK3R1 protein and Galangin complex stabilized by one hydrogen bond at Gln497 and two hydrophobic bonds at Tyr 508 and Ile 524 positions. Coumestrol formed two hydrogen bonds at Gln 501, Ser 505, one pi-pi-T shaped at Tyr 508 and one Pi-alkyl bond at Ile 524 positions. The positive control Wortmannin formed two hydrogen bonds at Gln 497 and Asn 527 with two hydrophobic interactions, Pi-Pi Stacked and Pi-alkyl bond at Tyr 504 position (Fig 9). In the VEGFA protein, sesamin formed seven hydrogens and four hydrophobic bonds, while galangin formed five hydrogens and one hydrophobic interaction. Cumestrol and quercetin formed four and eight hydrogen bonds, respectively (Fig 10).

### Chemo-informatics properties analysis

The ADMET properties, including physicochemical, lipophilicity, water-solubility, pharmacokinetics, drug-likeness, medicinal chemistry, and toxicity of the selected potent four compounds was tabulated in Table 7. The physicochemical properties of our targeted compound remained in expected value. The pharmacokinetics parameters show the high gastrointestinal (GI) absorption rate of the selected compounds. In terms of drug-like activity, none of the four compounds violates Lipinski’s rule of five. The water solubility reveals that three compounds are soluble in water, except alpha-mangostin are poorly soluble. The toxicity tests revealed that the human ether go-go-gene (hERG) is not inhibited by these compounds. The Ames test result data also express that only galangin compounds are not mutagenic compared with others. All the compounds show a negative response in skin sensitization and hepatotoxicity properties which is a good sign of a predictable drug molecule.

**Table 7:** Chemo-informatics analysis of selected compounds.

ADMET Properties		Sesamin	*α*-Mangostin	Galanzin	Coumestrol	Wortmannin
Physicochemical properties	MW (g/mol)	354.35	410.46	270.24	268.22	428.43
	NHA	26	30	20	20	31
	NAHA	12	14	16	17	5
	HBA	06	06	5	5	8
	HBD	00	03	3	2	0
	MR	90.00	119.99	73.99	73.81	105.71
	TPSA (A2)	55.38	100.13	90.90	83.81	109.11
Lipophilicity	iLOGP	3.46	4.14	2.08	1.80	2.70
	XLOGP3	2.68	6.27	2.25	2.76	1.18
	WLOGP	2.57	5.09	2.58	3.10	2.54
	MLOGP	1.98	2.19	0.52	1.76	0.94
	Silicos-IT Log P	3.25	5.52	2.52	2.88	3.64
	Consensus Log	2.79	4.64	1.99	2.46	2.20
Water Solubility	ESOL Log S	-3.93	-6.35	-3.46	-3.87	-3.10
	ESOL Class	Soluble	Poorly Souble	Soluble	Soluble	Soluble
	ALI Log S	-3.50	-8.16	-3.79	-4.18	-3.07
	ALI Class	Soluble	Poorly Souble	Soluble	Moderately soluble	Soluble
	Silicos-IT Log S	-4.60	-6.14	-4.40	-5.03	-5.15
	Silicos-IT Class	Moderately soluble	Poorly Souble	Moderately soluble	Moderately soluble	Moderately soluble
Pharmacokinetics Properties	GI absorption	High	High	High	High	High
	CYP1A2 inhibitor	No	No	Yes	Yes	No
	CYP2C19 inhibitor	Yes	No	No	No	No
	CYP2C9 inhibitor	No	yes	No	No	No
	CYP2D6 inhibitor	Yes	No	Yes	Yes	No
	CYP3A4 inhibitor	Yes	No	Yes	No	No
	Log Kp (skin permeation)	-6.56cm/s	-4.35 cm/s	-6.35 cm/s	-5.98 cm/s	-8.08 cm/s
Druglikeness	Lipinski	Yes; 0 violation	Yes; 0 violation	Yes; 0 violation	Yes; 0 violation	Yes; 0 violation
	Ghose	Yes	Yes	Yes	Yes	Yes
	Egan	Yes	Yes	Yes	Yes	Yes
	Bioavailability Score	0.55	0.55	0.55	0.55	0.55
Medicinal Chemistry	Pains	0 alert	0 alert	0 alert	0 alert	0 alert
	Leadlikeness	No;1 violation: MW>350	No;2 violation: MW>350, XLOGP3>3.5	Yes	Yes	No;1 violation: MW>350
	Synthetic accessibility	4.12	3.91	3.12	3.16	5.66
Toxicity	Ames test	Yes	Yes	No	Yes	Yes
	hERG I inhibitor	No	No	No	No	No
	hERG II inhibitor	No	Yes	No	No	No
	Skin Sensitisation	No	No	No	No	No
	Hepatotoxicity	No	No	No	No	No

### Potential validation targets using FI dataset and gold benchmark databases

**Fig 11:**
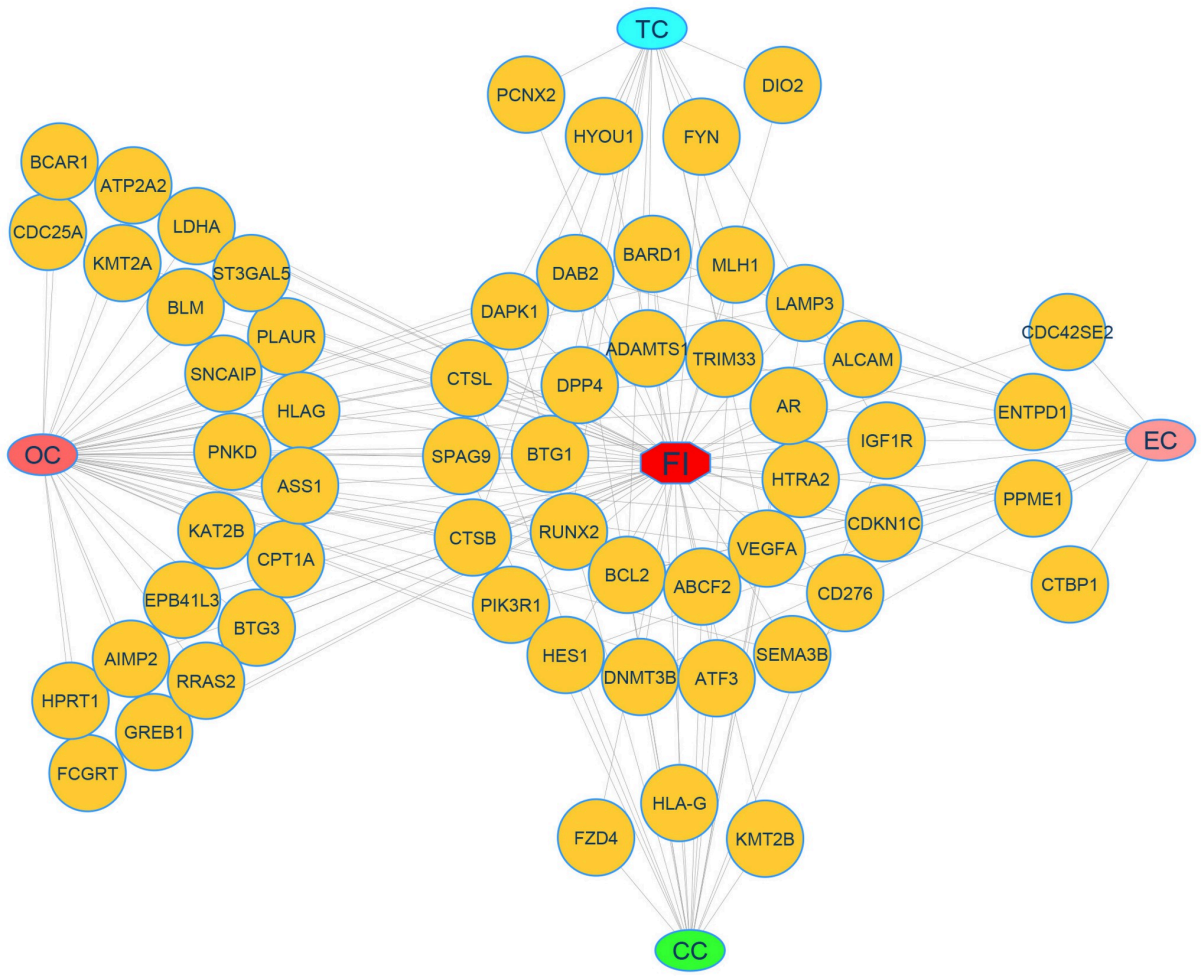
Gene-disease association analysis of DEGs of 4 cancerous risk factors with FI using OMIM and DisGNET databases. Ellipse-shaped nodes represent risk factors, and round-shaped nodes represent DEGs. Deep-green color and Round-shaped indicates common genes between FI and four cancerous risk factors.

To prove the common DEGs linked with FI, we added another dataset (GSE16532) for FI expression. The validation of the FI dataset is an Expression profiling array of data based on endometrium biopsy tissues from 4 infertile patients and 5 fertile women during the mid-secretory phase (LH +7) [3]. The suggested method then uses two gold-standard databases, such as Online Man Mendelian Heritage (OMIM) and DisGeNET as well as literature to authenticate the genes found in our research that indicate potential disease risk. We examined the overlap between all DEGs and a gene validation expression dataset. We found 16 DEGs correlated with each risk factors, including FI shared 9 DEGs with OC and 4 DEGs with EC, while TC and CC shared 21 DEGs, respectively. To verify our established findings, we have also incorporated Online Mendelian Inheritance for Man (OMIM) and DisGeNET databases to validate identified drug-gene associations [75]. As seen in Fig 11 genes linked with CC, OC, TC, and EC are likely to positively co-related with FI. Overall, our results fill in significant deficiencies in our knowledge of FI pathobiology and could open up new directions for establishing mechanistic correlations between FI and various cancerous risk factors.

## Discussion

Network-based bioinformatics and in- silico approaches offer more outstanding guidance for solid interaction and give a systemic view of mechanistic role in disease development. These studies can uncover new connections between the FI and other likelihood factors. Therefore, we analyzed dysregulation in the FI patients’ gene expression, molecular pathways, ontologies, and PPIs. Our results found multiple essential genes to determine the FI’s treatment objectives. Aside from that, our research identified and explained numerous metabolic roles associated with these genes. Our study of gene expression has shown that FI is closely linked to endometrial cancer (97 genes), ovarian cancer (211 genes), cervical cancer (87 genes), and thyroid cancer (33 genes) with sharing a maximum gene list. We created and examined the PPI linkage to recognize the central mechanism behind the FI better.

Additionally, diseases-associated genes play a critical role in human interaction via the pathways. In this study, we identified several significant pathways linked to cancer progressions such as cancer proteoglycans, TNF signalling pathway, ovarian infertility genes, diseases of signal transduction, focal adhesion, and notch signalling pathway. Several gene ontologies classes were also discovered in our research, including actomyosin structure organization, integrin-mediated signalling pathway, cholesterol metabolic process, ATP metabolic process, and cellular response to cytokine stimulus which are closely related to the cancer progression. Transplantable tumors in mice undergo fast hemorrhagic necrosis through the TNF pathway which also plays a role in cell transformation, survival, proliferation and metastasis stage of tumor growth [76, 77]. Changes influence cancer pathogenesis in mechanisms involved in signal transduction such as cell differentiation, proliferation and cell death [78]. The cytokine pattern generated by tumor cells indicated that immune stimulation coincides and might provide new therapeutic options for cancer patients [79]. Moreover, dysfunctional cholesterol metabolism in women may play a role in certain types of infertility [80] and gynecological cancer [81].

In this study, statistical analysis findings were used to develop a PPI network based on the differentially expressed genes. Topological research techniques were used to find core proteins (i.e., hubs). These identified hubs proteins may be used as possible drug targets or biomarkers. Ten hub proteins were seen in the PPI network study (VEGFA, PIK3R1, BCAR1, AR, CPT1A, ACSL3, ACSL4, IGF1R, LPL, and HMGCR) are involved in the FI. Endothelial cells (EC) mature in response to vascular endothelial cell growth factor (VEGF2) via the VEGF2 receptor, which is activated by cyclooxygenase (COX), while COX2 has been attributed to the onset of a variety of cancers, predominantly colon cancer [82]. The insulin-like growth factor receptor (IGF-1R) is a tyrosine kinase that regulates cell proliferation and survival. It is thought that this receptor inhibition might be a potential target in cancer therapy [58]. PI3K, a phosphoinositide 3-kinase, is upregulated in several human cancers, with oncogenic mutations discovered in the p110-alpha catalytic and p85-alpha regulatory subunits that serve as a docking site during the drug development phase [70, 83]. The serum anti-estrogen resistance protein 1 (BCAR1 also known as p130-cas) is a molecular marker for pulmonary disease diagnosis and prognosis [84]. The variant of the androgen receptor (AR), especially AR-V7 that facilitates AR targeted drug resistance.

In castration-resistant prostate cancer, AR-V9 could be a critical component of therapeutic resistance (CRPC) [85]. In vivo, statins target HMGCR (HMG-CoA reductase) in breast cancer cells and they may also have an anti-proliferative property in HMGCR progressive tumors [86]. The presence of an active FAO is a significant indicator of radiation resistance in nasopharyngeal carcinoma (NPC). So, CPT1A targeting may be a good way to improve radiotherapy’s beneficial efficacy in patients with nasopharyngeal carcinoma (NPC) [87]. A family of enzymes catalyses the activation of fatty acids by adding coenzyme A named the acyl CoA synthetase ligases (ACSL) showing more aggressive cancerous features [88].

The hub proteins we have identified in this study play a significant role in infertility and cancer biology. However, the molecular mechanism of FI and its link to the cancerous risk factor is controversial. Besides, several proteins are being explored as therapeutic approaches. This helps our network system to define genes of interest in our disease progression with a specific function. Then we selected VEGFA and PIK3R1 proteins for the docking approach based on the Maximal Clique Centrality (MCC) value of the cytohubba plugin and literature analysis indicates that VEGFA and PIK3R1 most common genes responsible for the progression of several cancer, including Endometrial cancer [62, 63], Ovarian cancer [23, 64], Cervical cancer [24, 65] and Thyroid cancer [66, 67]. Several frequent mutations, including Y504D, Q552K, I559N, D560Y, N564D, D569Y, R574T, T576del, W583del, N595K, and N600H mutants may regulate the iSH2 domain for the creation of multiple cancer like breast, endometrium and ovarian cancer particularly at amino acids 456-469 and 564-575 [70, 72–74] in PIK3R1 protein. In our analysis, we did not include the mutated form of PIK3R1 protein.

Further, selected phytoestrogenic compounds were used before as a therapeutic approach in several cancer treatments. Additionally, ADMET properties show a positive sign as a drug compound for treating the mentioned disease in our study.

Molecular modelling has become an integral part of modern drug development networks especially docking study [89]. Furthermore, time and laboratory costs can be reduced dramatically by reducing the choices of drug molecules in the silico based drug development phase [90]. Besides, the investigator can draw the binding insights into the target protein of the drug molecules and rationally define treatment objectives. However, of the 27 phytochemical compounds evaluated, the peak four are chosen based on their highest docking score compared to positive control inhibitors bevacizumab and wortmannin and found that sesamin, galangin, and coumestrol could be used as promising inhibitors against VEGFA and PIK3R1 proteins.

The three compounds have chosen exhibit strong non-covalent interactions with other predicted binding site residues by the CASTp server, including Lys 506, Ile 509, Tyr 504, Tyr 508, Ile 524, Tyr 528, Met 525, Lys 532 residues at the iSH2 domain of the B chain of PIK3R1 protein. All compounds that bind to the catalytic site of PIK3R1 protein and the same binding pockets ignoring the mutation region were found in our investigation. On the other hand, Glu 64, Ser 50, Cys 68, and Ile 46 were the active site of the VEGFA protein, where the top three compounds were bound. Sesamin can be used as an important therapeutic agent to prevent or treat different of types cancers [91]. This result indicates that sesamin inhibits cervical cancer cell proliferation via the induction of PTEN-mediated apoptosis [92]. A recent study concluded that, sesamin destroy thyroid cell expansion and initiates apoptosis by hindering STAT-3 translocation [93]. A recent study demonstrated that natural antioxidants, including sesamin, along with galangin, serve as a drug compound to prevent Covid-19 severity [94]. Shu Lio et al. showed that Coumestrol, not only has an inhibitory impact in terms of growth of three cancerous cell line but also serve as a cell penetrable CK2 inhibitor with submicromolar IC50 and a new ATP competitive [95]. Coumestrol suppresses proliferation of ES2 human epithelial ovarian cancer cells via PI3K and ERK1/2 MAPK pathways. These findings indicate that coumestrol showing an anti-cancer effect through the direct targeting of haspin kinase [96, 97]. H. Huang et al. established that galangin showed anti-cancerous activity via p53-dependent pathway; as a result, it can be used for the treatment purposes of platinum-resistant ovarian cancers in humans [98]. This result demonstrated that galangin could be used as an effective adjuvant to increase the efficacy of chemotherapeutic agents in several cancer treatments [99].

In the biopharmaceutical sector, computational biology techniques enhanced the drug discovery process not only to find but also to develop lead compounds against disease [100]. The physicochemical and drug-likeness properties of any compounds were determined by ADMET analysis in a productive and cost-saving approach way [101]. ADMET testing gives a good perception of potential drug applicants. A potential drug should have an optimum molecular weight between 150 and 500 g/mol (Dalton), the higher number of heavy atoms (NHA) and higher number of heavy aromatic atoms (NAHA), hydrogen bonding acceptor (HBA) ≤ 10, hydrogen bonding donor (HBD) ≤ 5, TPSA (topological polar surface area) around 20 to 130 Å2, and molar refractory (MR) range of 40 to 130 is a good indication of drug performance. Analysis of water solubility indicated that three compounds are soluble in water, except alpha-mangostin is poorly soluble. The medicinal chemistry properties of four compounds indicate that they have no PAINS (pan assay interference compounds) alert that refer to provide a strong propensity to attach to their targets with no false-positive results. Moreover, sesamin and alpha-mangostin infringe lead likeness properties, whereas coumestrol and galangin do not break these properties. The compounds studied in this research are moderately synthetic. (Assume value 1 for low and 10 for high synthetic accessibility). We examined the possible treatment mechanisms of the 27 phytochemicals in this network-based bioinformatics study against VEGFA and PIK3R1 hub proteins. By analyzing DEGs, molecular docking and ADMET properties, we suggest that identified hub proteins, especially VEGFA and PIK3R1 can be used as a target to treat infertility mediated cancer progression. Besides, the top 3 compounds, including sesamin, galangin and coumestrol, could contribute to new treatment techniques. Because previously, these compounds were used in several cancer treatments, but it was not treated in infertility mediated cancer progression. So, we investigate the transcriptomic data for identifying DEGs as the molecular target with therapeutic agents. However, further clarification is still needed for the mechanism of these active compounds through in vitro study which can facilitate the design of new drugs with a wide range of anti-cancerous activity.

## Conclusion

In this research, the microarray transcriptomic data were used to determine the genetic linkage of FI with multiple cancer. According to our findings, these network-based bioinformatics techniques can mention positive interaction of FI mediated cancer risk factors. We identified several mechanistic pathways and gene ontology biological processes like TNF signalling pathway, signal transduction, cholesterol metabolic process and cellular response to cytokine stimulus which provide a clear view of the genotypic link among multiple cancers and FI. The study of gene expression has also established ten hub proteins which significantly contribute to FI and cancer progression; among them VEGFA and PIK3R1 are the most significant for disease progressions. Molecular docking and ADMET properties against VEGFA (PDB ID: 1FLT) and PIK3R1 (PDB ID: 5M6U) showed sesamin, galangin, and coumestrol were the top three compounds that might play a role on therapeutic target in the treatment of infertility and infertility mediated cancer progression. This approach permits us to determine how their interactions can better understand FI processes from a mechanical perspective with cancer. We recommended that our identified pathway, hub proteins, and phytocompounds serve as new targets and therapeutic interventions for accurate diagnosis and treatment of multiple diseases. Further molecular dynamic simulation and clinical/in vitro study must be needed to confirm the activity of compounds, along with the co-relation of DEGs between FI and the formation of several cancers.
